# Quantitative evaluation of 3D-printed physiological visualization tools in enhancing interns’ knowledge retention and application

**DOI:** 10.3389/fmed.2026.1792731

**Published:** 2026-04-24

**Authors:** Qiongting Luo, Wenwen Hou, Xiaofen Yu, Xinyu Wang, Zheng Wang

**Affiliations:** Hangzhou Medical College (Affiliated People’s Hospital of Hangzhou Medical College, Zhejiang Provincial People’s Hospital), Hangzhou, China

**Keywords:** 3D intelligent printing technology, anatomical visualization, knowledge retention, medical curiosity, nursing interns, physiology teaching, self-efficacy, transformative learning readiness

## Abstract

**Background:**

Traditional physiology teaching relies on 2D materials and static specimens, making it difficult to intuitively present complex anatomical structures and physiological mechanisms. 3D Intelligent Printing Technology (3DIPT) has demonstrated application value in surgical training, but its use in physiology education remains underexplored.

**Methods:**

A randomized controlled trial (RCT) was conducted, enrolling 120 undergraduate nursing interns who were randomly divided into a control group (traditional teaching) and an observation group (3DIPT-assisted teaching) with a 6-month intervention period. The observation group used 3D-printed models of key nursing-relevant organs; this paper partially presents those of the ovary, uterus, stomach, prostate, and kidney for clinical education and connected learning. Outcome measures included scores on physiology-related knowledge (nurse licensing examination simulation), Social Medical Curiosity (SMC), self-directed learning ability, mobile learning willingness, and Medical Students’ Transformative Learning Readiness (MSTLR).

**Results:**

After the intervention, the observation group showed significantly higher scores than the control group in physiology knowledge (77.30 ± 9.65 vs. 67.36 ± 9.55, *p* < 0.01), SMC (24.90 ± 4.7 vs. 23.57 ± 3.40, *p* = 0.0395), autonomous learning ability (118.95 ± 3.15 vs. 117.10 ± 3.56, *p* = 0.0391), mobile learning willingness (126.60 ± 10.35 vs. 116.40 ± 10.20, *p* = 0.0268), and MSTLR (61.50 ± 5.35 vs. 56.10 ± 5.20, *p* = 0.0223).

**Conclusion:**

3DIPT-assisted teaching can effectively improve nursing interns’ mastery of physiology knowledge and core competencies such as medical interest and autonomous learning. It provides an intuitive visualization tool for physiology education and holds significant potential for advancing basic medical teaching reform.

## 1 Introduction

In recent years, the application of 3D intelligent printing technology in the medical field has expanded from preoperative planning and personalized implant manufacturing to surgical simulation and skills training. Previous studies by our team have verified the effectiveness and reliability of 3D intelligent printing models in simulating complex surgeries such as Nissen fundoplication, robot-assisted partial nephrectomy, and laparoscopic pancreaticojejunostomy (1–3). These studies have shown that 3D intelligent printing models can significantly improve surgeons’ surgical proficiency and decision-making capabilities through highly simulated anatomical structures and operational feedback, providing a new solution for clinical skills training (4, 5). The applications of virtual visualization and 3D printing in anatomical education have been widely reported internationally and domestically (6–10) with standardized processes for converting clinical imaging data into 3D printed anatomical models having been established (8). Technological innovations represented by 3D printing have greatly promoted the reform of anatomical teaching (7), and randomized controlled trials have confirmed that image-based interactive 3D modules can significantly improve students’ mastery of anatomical knowledge (10). However, most studies have focused on the single field of anatomy, and the integration of 3D visualization tools with physiology teaching and clinical practice, especially in nursing education, remains to be further explored. Traditional physiology teaching relies on 2D images, textual descriptions, and static specimens, making it difficult to intuitively present multi-system physiological interactions, normal-pathophysiological differences, and physiological adaptation mechanisms under special environments (11, 12). In large-scale courses, student-centered active learning models are difficult to implement due to the lack of personalized teaching aids (13), and although existing teaching reform strategies propose the concept of “student-centeredness” (14), traditional teaching aids fail to meet the demands of dynamic visualization and practical exploration, resulting in a gap between teaching effects and objectives. Notably, cutting-edge research in physiology education has clearly identified the key role of visualization tools in enhancing students’ understanding of abstract content such as cross-system physiological regulation and molecular physiological mechanisms (15–17). For example, studies in the Frontiers series have revealed teaching difficulties in complex mechanisms such as gut microbiota-host physiological interactions and circadian rhythm regulation (16, 17), while multiple papers in Advances in Physiology Education have systematically elaborated on the implementation barriers and optimization paths of active learning models in large-scale courses (11–14). Meanwhile, individual factors of medical students, such as medical interest (18, 19), autonomous learning ability (20), mobile learning willingness (21), and transformative learning readiness (22), have also been proven to be closely related to teaching effects, providing multi-dimensional indicators for evaluating the effectiveness of 3D intelligent printing-assisted teaching. Given the successful experience of 3D intelligent printing in surgical training and the urgent need for dynamic visualization tools in physiology education, this study aims to explore the application potential of 3D intelligent printing technology in physiology teaching. By constructing multi-dimensional 3D intelligent printing models (uterus, stomach, kidney, bladder/prostate, etc.) and integrating them with the routine teaching of undergraduate nursing students during their internships, multi-dimensional evaluations including nurse licensing examination knowledge points, medical interest, autonomous learning ability, mobile learning willingness, and transformative learning readiness were conducted to address the pain points in traditional physiology teaching and provide new visualization tools and teaching models for basic medical education including physiology.

## 2 Materials and methods

### 2.1 Study subjects and platform

1.1 This RCT was conducted at a provincial hospital in China from February 2025 to August 2025. A total of 132 undergraduate nursing interns were enrolled. To ensure scientific rigor and baseline balance between groups, a strict random allocation protocol was adopted. Sample size estimation was performed using SPSS 23.0 statistical software, and statistical descriptions included constituent ratios, mean ± standard deviation, and other indicators. Let the total sample size be N, the sample size of the control group be n1, and the sample size of the observation group be n2, i.e., *N* = n1 + n2; the sample sizes of the two groups were allocated at a 1:1 ratio (k = n1:n2 = 1). Referring to similar studies and pre-experimental data, the standard mean difference *σ* was set to 3.5, the mean difference between the two groups *ε* was 2.1, *α* = 0.05, and *β* = 0.2. Calculated according to statistical formulas, the minimum required sample size for each group in this study was 60 cases. Considering potential sample loss during the study (such as irregular questionnaire filling and loss to follow-up), the sample size was expanded by approximately 10% to account for attrition. Finally, 66 cases were assigned to both the control group and the observation group, with a total sample size of 132. Of the 132 enrolled interns, 12 were excluded due to attrition, leaving 120 cases (60 per group) for the final analysis.

*Inclusion criteria*: (1) Age ≥ 20 years; (2) Completed undergraduate theoretical learning at school, currently in the final year of internship and entering the clinical internship stage; (3) Understanding of the study purpose and voluntary participation.

*Exclusion criteria*: (1) Interruption of internship due to special circumstances; (2) Interns who took consecutive leave of more than 3 days or cumulative leave of more than 7 days during the internship period; (3) Participation in training of other teaching methods; (4) Incomplete data.

### 2.2 Data collection methods

Multiple data collection methods including questionnaires, skill assessments, self-assessment reports, and knowledge tests were adopted to comprehensively evaluate the effectiveness of the 3DIPT teaching method in improving undergraduate nursing interns’ mastery of knowledge points related to the nurse licensing examination, medical interest, autonomous learning ability, mobile learning willingness, and transformative learning readiness. In addition, during data collection, emphasis was placed on ensuring data accuracy and reliability, protecting participants’ privacy and rights, and minimizing bias and errors.

### 2.3 Teaching intervention

- *Control group*: Interns received traditional internship teaching based on the standard Clinical Nursing Internship Manual, with teaching methods including traditional bedside teaching, case discussions, and theoretical lectures.
- *Observation group*: On the basis of routine internship teaching, the observation group adopted 3DIPT-assisted teaching for 6 months, with the implementation steps as follows:

### 2.4 Platform construction

The teaching team and interns jointly selected core physiological organs that were either of interest or difficult to learn. Materials from the Visible Human Project were used as the anatomophysiological basis, which has been well-recognized as a gold standard for standardized anatomical and physiological data in medical education and 3D model construction (6, 23, 24). Combined with the mature experience of anatomical visualization from the Korean Visible Human Project adapted for Asian population characteristics (25) and web-based 3D visualization applications (26, 27), we retrieved MDCT image data from a tertiary hospital in the province where the internship was conducted using Zhongnan e3D Digital Medical Virtual Software V17.06 (China) for three-dimensional reconstruction, ensuring the accuracy and clinical relevance of the models for Chinese nursing interns.

#### 2.4.1 Interactive discussions

For complex organs, 15–20 min WeChat group video conferences were held, involving rotating department teachers, 3D printing professionals, and interns to analyze anatomical structures, model printing quality, and key clinical practice points. Notably, the effectiveness of 3D-printed simulators in surgical training has been widely verified, providing tangible and realistic models that enhance skill acquisition.

#### 2.4.2 Model printing

**Figure 1:**
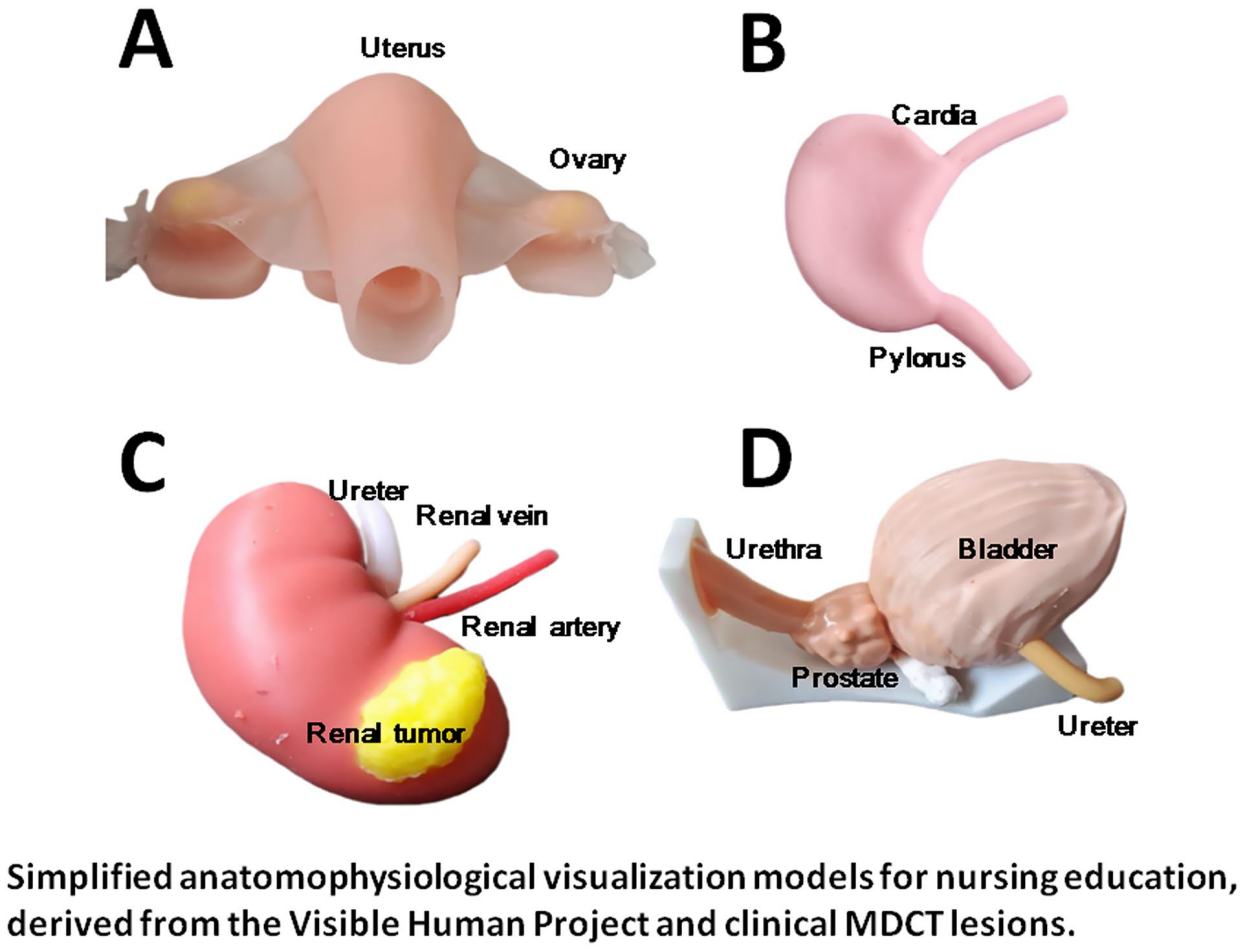
3D-printed organ models for physiology teaching. These models replicate the core anatomical structures and spatial relationships of key organs, facilitating the visualization of abstract physiological mechanisms. **(A)** Uterus and ovaries: To illustrate the basic anatomy of the female reproductive system. **(B)** Stomach: To demonstrate the gross structure of the stomach (including the fundus and pylorus), assisting in the learning of gastric motility, acid secretion, and digestive physiology. **(C)** Kidney with renal tumor: To replicate the renal vascular distribution and renal hilum, clarifying the anatomical relationship between the renal tumor and the urinary system. **(D)** Bladder, prostate, and urethra (with ureters): To present the anatomical structure and spatial relationship of the lower urinary tract, supporting the understanding of urine storage, micturition reflex, and related physiological functions.

**Figure 2:**
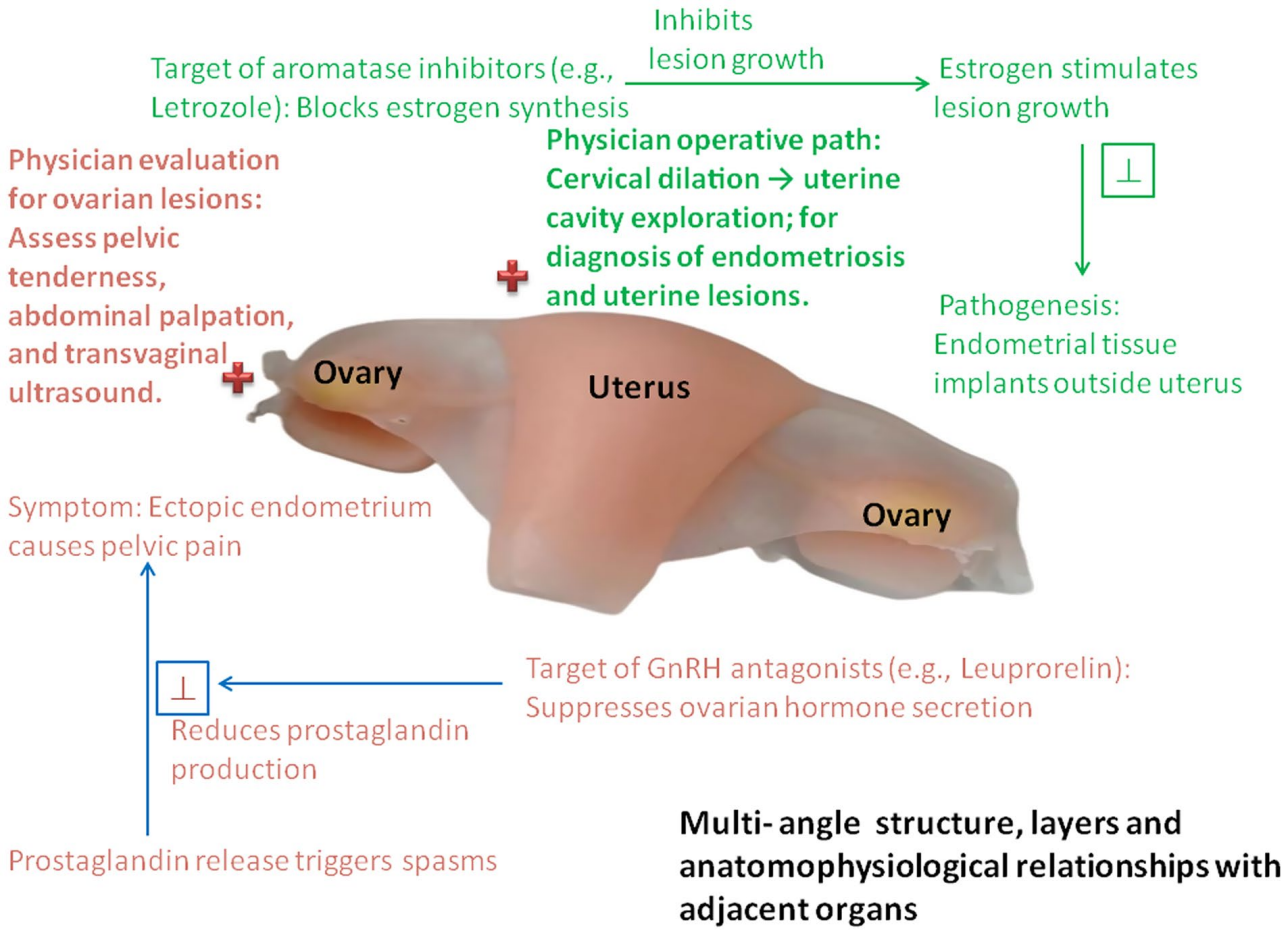
In-depth functional anatomical demonstration of the uterus-ovary complex: a representative example from the multi-organ models in Figure 1. This enhanced 3D-printed model, derived from one of the multi-organ models presented in Figure 1, provides a detailed illustration of the uterus-ovary-fallopian tube complex. It highlights multi-angle structural layers, key functional units, and endophysiological relationships with adjacent organs, further integrating pathological mechanisms, clinical symptoms, and therapeutic targets. This in-depth demonstration transforms static anatomical visualization into a dynamic teaching tool, enabling medical students and interns to achieve a deeper, clinically oriented understanding of anatomy, physiology, and disease processes.

Ultimaker Cura 4.4.1 open-source slicing software (United States) was used to analyze lesions and ducts, generate G-code, and Zhongrui Zhichuang SL600 stereolithography rapid prototyping equipment (China) with a composite material of soft resin and hard resin was used to print organ models with anatomical layers and lesion characteristics. The models were dyed for teaching purposes (see Figures 1, 2).

## 3 Evaluation tools

### 3.1 Main evaluation scale

The primary outcome was the score of a self-designed simulation test paper on physiology-related knowledge points of the nurse licensing examination, reflecting interns’ ability to apply theoretical knowledge to clinical practice.

### 3.2 Secondary evaluation scales included

#### 3.2.1 Medical curiosity assessment tool

The Chinese version of the scale translated and revised by Yang Tiantian, including 2 dimensions [Intellectual Medical Curiosity (IMC) and Social Medical Curiosity (SMC)] with a total of 10 items. Each item was scored on a 7-point Likert scale (1 = “Completely inconsistent” to 7 = “Completely consistent”). The overall Cronbach’s *α* coefficient of the scale was 0.852, and the Cronbach’s α coefficients of each dimension were 0.796 and 0.866, respectively, which is suitable for evaluating the medical curiosity of Chinese undergraduate nursing students (19). Among them, the Intellectual Medical Curiosity (IMC) dimension was excluded due to insignificant changes during the learning period (18), and only the SMC scoring scale was used in this study.

#### 3.2.2 Medical students’ self-directed learning ability assessment tool

The Medical Students’ Self-Directed Learning Ability Assessment Scale constructed by Wang Xiaodan et al., which covers 4 dimensions (learning goal setting, learning plan execution, learning resource utilization, and learning effect reflection) with a total of 28 items, using a 5-point Likert scale (1 = “Completely inconsistent” to 5 = “Completely consistent”). Each item was scored from 1 to 5, and the total score ranges from 28 to 140, with higher scores representing stronger self-directed learning ability. The overall Cronbach’s *α* coefficient of the scale was 0.893, and the Cronbach’s *α* coefficients of each dimension ranged from 0.782 to 0.861, which is suitable for the quantitative evaluation of medical students’ self-directed learning ability (20).

#### 3.2.3 Medical students’ Mobile learning willingness assessment tool (MLWS-MS)

The Chinese version of the Medical Students’ Mobile Learning Willingness Scale (MLWS-MS), translated, revised, and validated by Zheng Xiaoying et al., includes 4 dimensions (mobile learning cognition, mobile learning ability, mobile learning needs, and mobile learning ethics) with a total of 44 items. Each item was scored on a 5-point Likert scale (1 = “Completely disagree” to 5 = “Completely agree”). The total score ranges from 44 to 220, with higher scores indicating stronger willingness to use mobile learning. The overall Cronbach’s *α* coefficient of the scale was 0.876, and the Cronbach’s α coefficients of each dimension ranged from 0.765 to 0.843, which is suitable for evaluating the acceptance and use willingness of mobile learning among Chinese medical students (21).

#### 3.2.4 Medical students’ transformative learning readiness (MSTLR) assessment tool

The Medical Students’ Transformative Learning Readiness Scale (MSTLR) compiled by He and Deng et al. includes 4 dimensions (critical thinking, knowledge transfer ability, innovative awareness, and practical application willingness) with a total of 25 items, using a 5-point Likert scale (1 = “Completely not possessed” to 5 = “Completely possessed”). Each item was scored from 1 to 5, and the total score ranges from 25 to 125, with higher scores representing higher transformative learning readiness. The overall Cronbach’s α coefficient of the scale was 0.887, and the Cronbach’s α coefficients of each dimension ranged from 0.773 to 0.859, which can effectively evaluate the readiness of medical students to transform theoretical knowledge into practical abilities (22).

## 4 Data collection and statistical analysis

Before and after the 6-month intervention, data were collected through questionnaires, self-assessment reports, and knowledge tests. All data were analyzed using SPSS 29.0 software (SPSS Inc., Chicago, IL, United States). Categorical variables were presented as *n* (%), and compared using Pearson’s chi-square test or Fisher’s exact test (when sample size < 40 or theoretical frequency T < 1). Normality test of continuous variables was performed using the Shapiro–Wilk test; normally distributed data were presented as mean ± standard deviation and compared using independent samples *t*-test, while non-normally distributed data were presented as median (25th percentile, 75th percentile) and compared using the Wilcoxon rank-sum test. A *p* value < 0.05 was considered statistically significant.

### 4.1 Ethical approval

This study was approved by the Ethics Review Board of Hangzhou Medical College (Approval No.: LL2025-011). All procedures involving human participants in this study were in accordance with the Declaration of Helsinki and its subsequent amendments or similar ethical standards. All participants provided written informed consent, and data were collected anonymously to protect privacy. No patients were involved, and there was no risk of physical or psychological harm.

## 5 Results

### 5.1 Baseline characteristics

There were no statistically significant differences in baseline characteristics (age, gender, ethnicity, only-child status, GPA) between the two groups (all *p* > 0.05), indicating similar baseline conditions (Table 1).

**Table 1:** Baseline characteristics of participants in the control and observation groups.

Parameters	Traditional teaching group (*n* = 60)	3DIPT teaching group (*n* = 60)	Test statistic (*t*/*χ^2^*)	*p* value
Age (years)	21.32 ± 0.90	21.45 ± 0.86	0.809	0.420
Gender (M/F)	15 (25.00%/45 (75.00%)	16 (26.67%)/44 (73.33%)	0.440	0.507
Ethnicity (Han)	53 (88.33%)	54 (90.00%)	0.859	0.354
Residence (Urban/Rural)	16 (26.67%)/44 (73.33%)	17 (28.33%)/43 (71.67%)	0.412	0.521
Only-child (Yes)	14 (23.33%)	15 (25.00%)	0.448	0.503
Undergraduate GPA	4.80 ± 0.71	4.76 ± 0.68	0.315	0.753

Continuous variables are presented as mean ± standard deviation and compared using independent samples *t*-test; categorical variables are presented as *n* (%) and compared using Pearson’s chi-square test.

### 5.2 Comparison of outcome indicators before and after intervention

**Figure 3:**
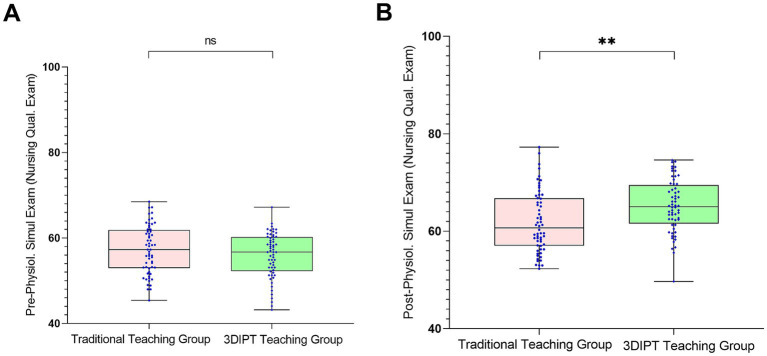
Comparison of scores of the simulation test paper on physiology-related knowledge points of the nurse licensing examination between the two groups before **(A)** and (B) after intervention. Data are mean ± SD, **p < 0.01 vs control. ns: not significant, **: *p* < 0.01.

**Figure 4:**
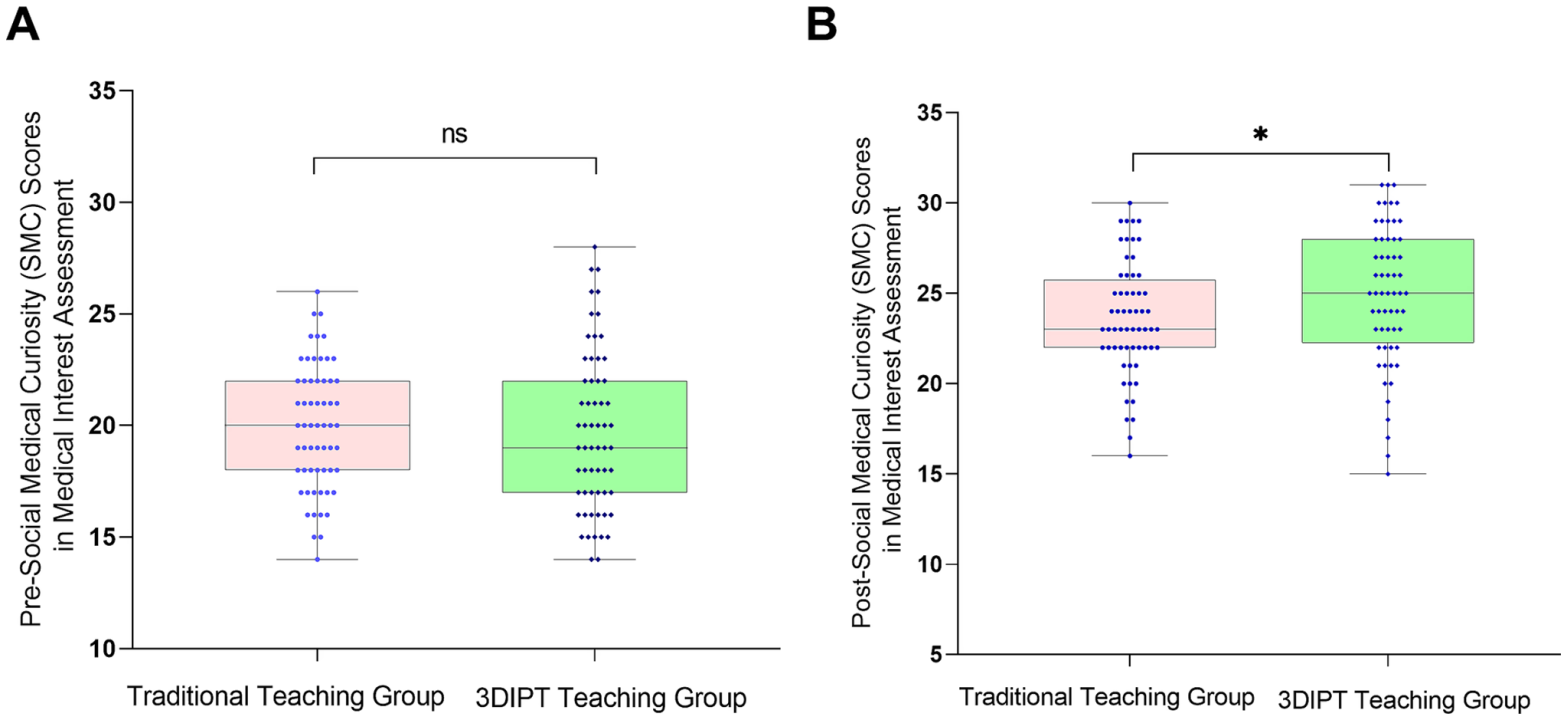
Comparison of social medical curiosity (SMC) scores between the two groups before **(A)** and **(B)** after intervention. Data are presented as mean ± standard deviation. *p < 0.05 compared with the control group after intervention.

**Figure 5:**
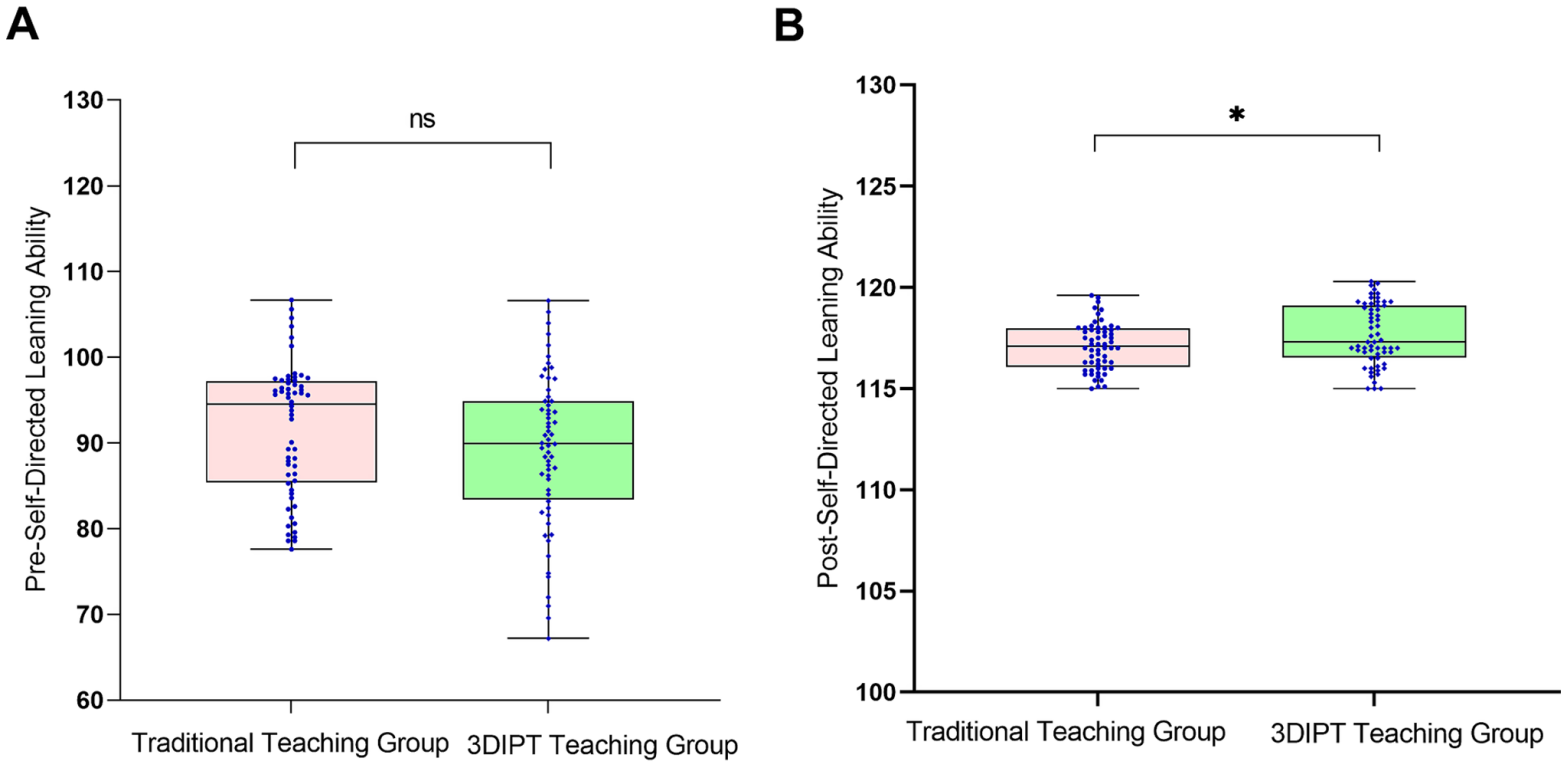
Comparison of autonomous learning ability scores between the two groups before **(A)** and **(B)** after intervention. Data are presented as mean ± standard deviation. *p < 0.05 compared with the control group after intervention.

**Figure 6:**
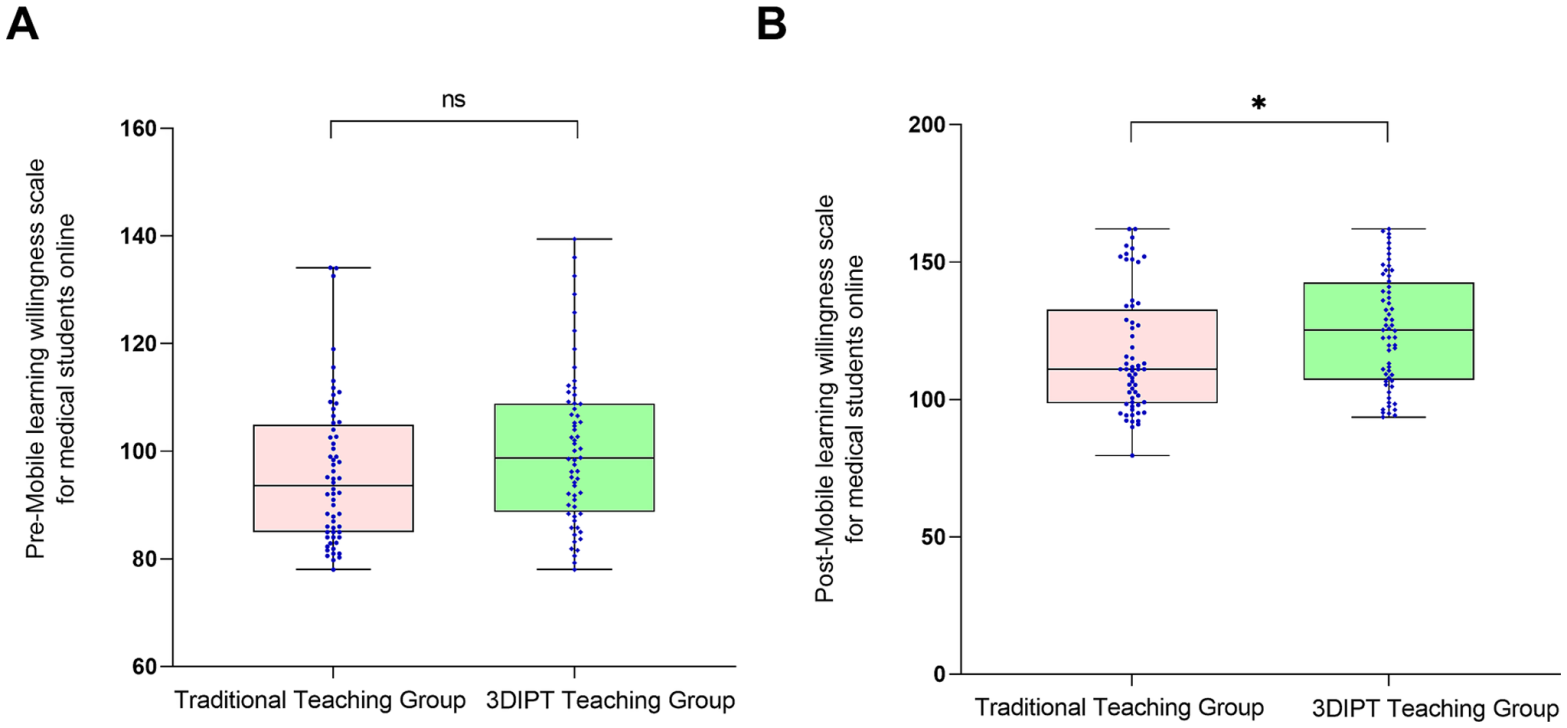
Comparison of mobile learning ability scores between the two groups before **(A)** and **(B)** after intervention. Data are presented as mean ± standard deviation. *p < 0.05 compared with the control group after intervention.

**Figure 7:**
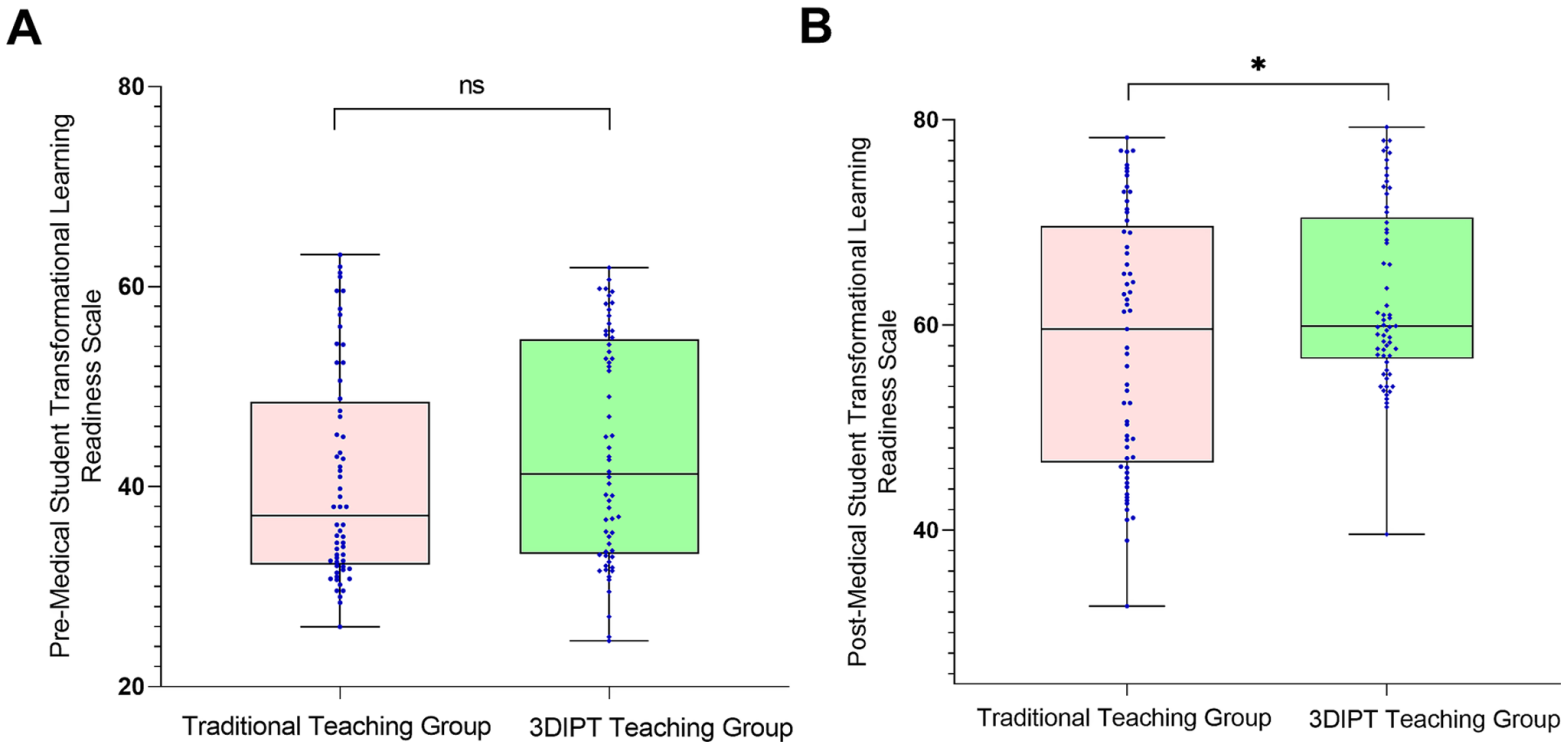
Comparison of medical students’ transformative learning readiness (MSTLR) scores between the two groups before **(A)** and **(B)** after intervention. Data are presented as mean ± standard deviation. *p < 0.05 compared with the control group after intervention.

- Score of the simulation test paper on physiology-related knowledge points of the nurse licensing examination: The scores were similar before intervention (59.10 ± 9.5 vs. 60.60 ± 8.8, *t* = 0.9983, *p* = 0.3202). After intervention, the score of the observation group was significantly higher (77.30 ± 9.65 vs. 67.36 ± 9.55, *t* = 6.032, *p* < 0.001) (Figure 3), reflecting a significant improvement in mastery of physiology knowledge points.
- Social Medical Curiosity (SMC) score: There was no statistically significant difference between the two groups before intervention (19.73 ± 3.62 vs. 19.93 ± 2.80, *t* = 0.3402, *p* = 0.7343). After intervention, the SMC score of the observation group was significantly higher than that of the control group (24.90 ± 4.7 vs. 23.57 ± 3.40, *t* = 2.082, *p* = 0.0395) (Figure 4), indicating an improvement in medical interest.
- Autonomous learning ability score: The scores were similar between the two groups before intervention (96.95 ± 2.55 vs. 96.45 ± 2.45, *t* = 1.323, *p* = 0.1883). After intervention, the score of the observation group was significantly higher (118.95 ± 3.15 vs. 117.10 ± 3.56, *t* = 2.087, *p* = 0.0391) (Figure 5), reflecting enhanced learning autonomy and initiative.
- Mobile learning ability score: There was no statistically significant difference before intervention (95.90 ± 4.45 vs. 100.50 ± 4.57, *t* = 1.730, *p* = 0.0863). After intervention, the score of the observation group was significantly higher (126.60 ± 10.35 vs. 116.40 ± 10.20, *t* = 2.242, *p* = 0.0268) (Figure 6), suggesting improved mobile learning ability.
- Medical Students’ Transformative Learning Readiness (MSTLR) score: The scores were similar before intervention (41.05 ± 3.42 vs. 44.50 ± 3.60, *t* = 1.648, *p* = 0.1019). After intervention, the score of the observation group was significantly higher (61.50 ± 5.35 vs. 56.10 ± 5.20, *t* = 2.315, *p* = 0.0223) (Figure 7), indicating enhanced transformative learning ability.

## 6 Discussion

This study expands the application of 3D printing technology from surgical simulation to the field of physiology teaching, which not only continues the research context of the team in the medical application of 3D printing (1–3) but also responds to the urgent need for dynamic visualization tools in physiology education (11, 12, 14). In teaching practice, 3D-printed models (uterus, stomach, kidney, bladder/prostate) can intuitively present cross-system physiological connections, addressing the cognitive bias of “organ isolation” in traditional teaching (15, 17). “Normal-pathological control models” (such as renal tumor models) help students distinguish structural and functional differences, deepening their understanding of pathophysiological mechanisms (28). In addition, modular 3D models are compatible with active teaching models such as Team-Based Learning (TBL), providing the possibility of personalized practice in large-scale courses (11, 12), which is highly consistent with the theoretical framework of teaching reform advocated by Advances in Physiology Education (14). Notably, multi-dimensional scale evaluation revealed that 3D printing-assisted teaching not only improved students’ comprehension of physiological mechanisms but also significantly enhanced their core clinical competencies including medical curiosity (18, 19), autonomous learning ability (20), mobile learning willingness (21), and transformative learning readiness (22). Notably, the educational value of 3D visualization tools for nursing students has been confirmed in previous studies, which found that blended learning with such tools can effectively improve nursing students’ mastery of anatomical knowledge (10). Our study further expanded this research to the integration of anatomy and physiology, and even to the connection with clinical practice, which is a further exploration on the basis of previous studies. In comparison with other 3D visualization technologies such as augmented reality and virtual anatomy dissection (7, 8), our 3D-printed physical models have the advantages of no equipment dependence and tangible interactive experience, which is more suitable for the clinical internship scene of nursing students and can better meet the needs of bedside teaching and case discussion. This finding can be deeply explained by the theories of cognitive load and embodied cognition: the 3D-printed visual models transform abstract anatomical structures and physiological mechanisms in traditional teaching into perceptible physical carriers, effectively reducing the extraneous cognitive load of nursing interns in knowledge acquisition and avoiding the excessive consumption of cognitive resources caused by the abstraction of 2D teaching materials (29). Meanwhile, the core logic of embodied cognition emphasizes the deep integration of bodily perception and cognitive processing (30); the 3D models provide interns with multi-dimensional perceptual and interactive experiences of anatomical structures, converting the learning of physiological knowledge from pure visual memory into embodied learning based on physical perception, thus facilitating the in-depth understanding of knowledge and the improvement of clinical application ability. Beyond cognitive and perceptual mechanisms, the significant improvements in students’ autonomous learning ability, medical curiosity and transformative learning readiness can be further elucidated by Bandura’s self-efficacy theory (31), which posits that perceived self-efficacy is a core driver of learning motivation, behavioral persistence and competency development in educational contexts. Traditional physiology teaching with abstract 2D materials often leads to low self-efficacy among nursing interns due to cognitive barriers and repeated learning frustrations, while 3D-printed tangible models offer concrete mastery experiences and vicarious learning opportunities (4), helping students build confidence in understanding complex physiological knowledge and applying it to clinical practice. This enhancement of self-efficacy further mediates the improvement of core learning competencies, which is consistent with recent medical education research confirming the critical mediating role of self-efficacy in linking innovative teaching tools to improved learning outcomes for medical students (1). This result indicates that 3D-printed models not only solve the teaching dilemma of abstract physiological mechanisms from cognitive and perceptual perspectives but also boost students’ learning motivation and self-confidence from the perspective of psychological efficacy, thus promoting the comprehensive development of nursing interns. This multi-dimensional mechanism further verifies the unique value of 3D printing technology in physiology education, providing stronger theoretical and practical support for the visualization innovation of basic medical education (37). In conclusion, the application of 3D printing technology in physiology teaching is expected to break through the limitations of traditional education and provide a new path for the innovative development of basic medical education (32). The preliminary exploration of this study also lays a foundation for subsequent large-scale teaching experiments, and in-depth research on the optimization of model design and teaching mode combination is worthy of further exploration (33, 34, 38, 39).

### 6.1 Limitations

This study has several limitations: (1) It is a single-center study, and the generalizability of the results is limited to other medical institutions in developing countries; (2) Due to the limited sample size, subgroup analysis based on gender, educational background, or career intentions was not performed; (3) The 6-month intervention period only reflects short-term effects, and long-term follow-up is needed to evaluate the sustained improvement of abilities; (4) Self-assessment scales may introduce response bias; future studies should integrate qualitative methods such as interviews and clinical observations for comprehensive analysis; (5) The intervention group received additional interactive support via WeChat video conferences with instructors and 3D printing experts, which may serve as a confounding variable. Thus, the improved learning outcomes cannot be solely attributed to the 3D-printed models, but may also be associated with enhanced teacher-student interaction and the interns’ participation in the conception and development of the 3D models that fostered their medical curiosity and autonomous learning capabilities. Future research should control for such confounding factors by providing equivalent interactive sessions for the control group. (6) The 3D-printed models in this study were purposefully simplified to match the key training points and examination syllabus for undergraduate nursing interns. Limited by the current immaturity of 3D printing technology and ongoing exploration of printing materials, the refinement of fine anatomical and physiological structures cannot yet reach the precise level of the Visible Human Project. Although such simplified models support embodied learning, enhance students’ professional identity, and help consolidate knowledge, the insufficient anatomical precision is still an obvious limitation of this study. With the continuous maturation of 3D printing technology and materials in the future, we will further improve the anatomical and physiological accuracy of the models (35, 36).

## 7 Conclusion

3DIPT-assisted teaching significantly improves the mastery of physiology knowledge points and other core competencies of medical talents among undergraduate nursing students during their internships, including medical interest, autonomous learning ability, mobile learning willingness, and transformative learning ability. It effectively addresses the need for visual and variable teaching models of physiology and different physiological states. With the transformation of clinical medical education towards competency-based training, 3DIPT has great potential in promoting the training of high-quality medical talents and improving clinical medical standards. Future multi-center, longitudinal studies are needed to verify the long-term effects of 3DIPT and optimize the teaching model for broader application in physiology learning.

## Data Availability

The original contributions presented in the study are included in the article/supplementary material, further inquiries can be directed to the corresponding authors.

## References

[1] Wang J Li L Zhang Y. The mediating effect of self-efficacy on the relationship between cancer early screening knowledge and screening behavior among medical students. Chin J Public Health. (2024) 40:612–6.

[2] Yang JT Luo P Wang Z Shen J. Simulation training of laparoscopic pancreaticojejunostomy and stepwise training program on a 3D-printed model. Int J Surg. (2022) 107:106958. doi: 10.1016/j.ijsu.2022.106958, 36283653

[3] Zhang Y Xia J Zhang J Mao J Chen H Lin H . Validity of a soft and flexible 3D-printed Nissen fundoplication model in surgical training. International Journal of Bioprinting. (2022) 8:546. doi: 10.18063/ijb.v8i2.546, 35669328 PMC9159478

[4] Wei F Xu M Lai X Zhang J Yiengpruksawan A Lu Y . Three-dimensional printed dry lab training models to simulate robotic-assisted pancreatojejunostomy. ANZ J Surg. (2019) 89:1631–5. doi: 10.1111/ans.15544, 31692187

[5] Yu H Yu T Wang J Wei F Gong H Dong H . Validation of a three-dimensional printed dry lab pancreaticojejunostomy model in surgical assessment: a cross-sectional study. BMJ Open. (2022) 12:e052295. doi: 10.1136/bmjopen-2021-052295, 35105574 PMC8808463

[6] Al-Redouan A Dudin A Urbanek AJ Olsson E Kachlik D. Visible human project based applications can prompt integrating cross-sectional anatomy into the medical school curriculum when combined with radiological modalities: a three-year cross-sectional observational study. Ann Anat. (2025) 257:152357. doi: 10.1016/j.aanat.2024.152357, 39577816

[7] Arráez-Aybar LA. Evolving anatomy education: bridging dissection, traditional methods, and technological innovation for clinical excellence. Anatomia. (2025) 4:9. doi: 10.3390/anatomia4020009

[8] Bork F Stratmann L Enssle S Eck U Navab N Waschke J . The benefits of an augmented reality magic Mirror system for integrated radiology teaching in gross anatomy. Anat Sci Educ. (2019) 12:585–98. doi: 10.1002/ase.1864, 30697948 PMC6899842

[9] Darras KE Spouge R Hatala R Nicolaou S Hu J Worthington A . Integrated virtual and cadaveric dissection laboratories enhance first year medical students' anatomy experience: a pilot study. BMC Med Educ. (2019) 19:366. doi: 10.1186/s12909-019-1806-5, 31590672 PMC6781397

[10] Sumner C Case SL Franklin S Platt K. Interactive, image-based modules as a complement to Prosection-based anatomy laboratories: multi cohort evaluation. JMIR Medical Education. (2026) 12:e85028. doi: 10.2196/85028, 41529253 PMC12848492

[11] Goodman BE Barker MK Cooke JE. Best practices in active and student-centered learning in physiology classes. Adv Physiol Educ. (2018) 42:417–23. doi: 10.1152/advan.00064.2018, 29972063

[12] Kibble JD Bellew C Asmar A Barkley L. Team-based learning in large enrollment classes. Adv Physiol Educ. (2016) 40:435–42. doi: 10.1152/advan.00095.2016, 27697956

[13] Griff ER. Changing undergraduate human anatomy and physiology laboratories: perspectives from a large-enrollment course. Adv Physiol Educ. (2016) 40:388–92. doi: 10.1152/advan.00057.2016, 27503898

[14] Goodman BE. An evolution in student-centered teaching. Adv Physiol Educ. (2016) 40:278–82. doi: 10.1152/advan.00056.2016, 27445274 PMC5504412

[15] Leulier F MacNeil LT Lee WJ Rawls JF Cani PD Schwarzer M . Integrative physiology: at the crossroads of nutrition, microbiota, animal physiology, and human health. Cell Metab. (2017) 25:522–34. doi: 10.1016/j.cmet.2017.02.001, 28273475 PMC6200423

[16] Li H Li K Zhang K Li Y Gu H Liu H . The circadian physiology: implications in livestock health. Int J Mol Sci. (2021) 22:2111. doi: 10.3390/ijms22042111, 33672703 PMC7924354

[17] Riedl RA Atkinson SN Burnett CML Grobe JL Kirby JR. The gut microbiome, energy homeostasis, and implications for hypertension. Curr Hypertens Rep. (2017) 19:27. doi: 10.1007/s11906-017-0721-6, 28316052 PMC5773096

[18] Bugaj TJ Schwarz TA Terhoeven V Nagy E Cranz A Friederich HC . Measuring an understudied factor in medical education—development and validation of the medical curiosity scale. Med Educ Online. (2023) 28:2198117. doi: 10.1080/10872981.2023.2198117, 37014965 PMC10075518

[19] Yang TT Wang Y Huang ZL Guo XB Zhang X Guo Y . Cross-cultural adaptation and psychometric validation of the medical curiosity scale among nursing undergraduates in China. J Nurs Sci. (2025) 40:75–8.

[20] Wang XD Tang GQ Wang SZ Ma JD Liu W Tian H . Construction of the self-learning ability scale for medical students. China J Health Psychol. (2014) 22:1034–7.

[21] Zheng XY Li DY Lü XL. Cross-cultural adaptation and psychometric validation of the Mobile learning willingness scale for medical students in China. J Nurs. (2025) 32:1–6.

[22] He JQ Yan WQ Deng RZ Chen SQ Dong XQ Ma X . Development and psychometric validation of the transformative learning readiness scale for medical students. Chinese Higher Medical Education. (2025) 8:43–6.

[23] Ackerman MJ. The visible human project. Information Services Use. (2022a) 42:129–36. doi: 10.3233/ISU-210145, 35600127 PMC9108582

[24] Ackerman MJ. The visible human project. Stud Health Technol Inform. (2022b) 288:134–40. doi: 10.3233/SHTI210988, 35102835

[25] Kim CY Chung MS Park JS. Visible Korean based on true color sectioned images for making realistic digital human, twenty years' record: a review. Surg Radiol Anat. (2024) 46:935–47. doi: 10.1007/s00276-024-03381-2, 38717503

[26] Borgbjerg J. Web-based imaging viewer for real-color volumetric reconstruction of human visible project and DICOM datasets. Clin Anat. (2021) 34:470–7. doi: 10.1002/ca.23712, 33347648

[27] Carroll MA Gluschitz S. "Constructing the body: the intersection of standardizing anatomy, illustration, and digitization". In: Decolonial Perspectives in Biomedical Sciences, Anatomical Education and Healthcare. Berlin: Springer (2026)

[28] van Mens TE Scheres LJ de Jong PG Leeflang MM Nijkeuter M Middeldorp S. Imaging for the exclusion of pulmonary embolism in pregnancy. Cochrane Database Syst Rev. (2017) 2017:CD011053. doi: 10.1002/14651858.CD011053.pub2, 28124411 PMC6464730

[29] Nagyné Elek R Haidegger T. Non-technical skill assessment and mental load evaluation in robot-assisted minimally invasive surgery. Sensors. (2021) 21:2666. doi: 10.3390/s21082666, 33920087 PMC8068868

[30] Mende MA Schmidt H. Psychotherapy in the framework of embodied cognition-does interpersonal synchrony influence therapy success? Front Psych. (2021) 12:562490. doi: 10.3389/fpsyt.2021.562490, 33828491 PMC8019827

[31] Bandura A. Self-efficacy: toward a unifying theory of behavioral change. Psychol Rev. (1977) 84:191–215. doi: 10.1037//0033-295x.84.2.191, 847061

[32] Blackwell JE Kingshott RN Weighall A Elphick HE Nash H. Paediatric narcolepsy: a review of diagnosis and management. Arch Dis Child. (2022) 107:7–11. doi: 10.1136/archdischild-2020-320671, 33975822

[33] Costello JP Olivieri LJ Krieger A Thabit O Marshall MB Yoo SJ . Utilizing three-dimensional printing technology to assess the feasibility of high-Fidelity synthetic ventricular septal defect models for simulation in medical education. World Journal of Pediatric and Congenital Heart Surgery. (2014) 5:421–6. doi: 10.1177/2150135114528721, 24958045

[34] Cui D Wilson TD Rockhold RW Lehman MN Lynch JC. Evaluation of the effectiveness of 3D vascular stereoscopic models in anatomy instruction for first year medical students. Anat Sci Educ. (2017) 10:34–45. doi: 10.1002/ase.1626, 27273896

[35] Manchenster KR Roberts D. From classroom to clinic: bridging the gap in nursing anatomy and physiology education. Nurse Educ Pract. (2024) 75:103870. doi: 10.1016/j.nepr.2023.103870 38129254

[36] Ramachandran V Wang R Ramachandran SS Ahmed AS Phan K Antonsen EL. Effects of spaceflight on cartilage: implications on spinal physiology. Journal of Spine Surgery. (2018) 4:433–45. doi: 10.21037/jss.2018.04.07, 30069539 PMC6046341

[37] Thammasitboon S Brand PLP. The physiology of learning: strategies clinical teachers can adopt to facilitate learning. Eur J Pediatr. (2022) 181:429–33. doi: 10.1007/s00431-021-04054-7, 33782760 PMC8821380

[38] Wang Z Wang XY Yu XF. Application of 3D printing surgical training models in the preoperative assessment of robot-assisted partial nephrectomy. BMC Surg. (2024) 24:167. doi: 10.1186/s12893-024-02456-6, 38807080 PMC11131263

[39] Yahiro DS Abrantes JCDS Magliano DC Mesquita CT. Creation of cardiac embryological models for 3D printing to teach anatomy and embryology. Arq Bras Cardiol. (2023) 120:e20220632. doi: 10.36660/abc.20220632, 37098991 PMC10124573

